# Acetylcholinesterase (AChE) Reversible Inhibitors: The Role of Oxamyl in the Production of Poisoned Baits

**DOI:** 10.3390/toxics10080432

**Published:** 2022-07-29

**Authors:** Alberto Biancardi, Cristina Aimo, Pierluigi Piazza, Federica Lo Chiano, Silva Rubini, Erika Baldini, Silvia Vertuani, Stefano Manfredini

**Affiliations:** 1 Experimental Zooprophylactic Institute of Lombardy and Emilia Romagna, Via A. Bianchi 9, 25124 Brescia, Italy; alberto.biancardi@izsler.it (A.B.); cristina.aimo@izsler.it (C.A.); pierluigi.piazza@izsler.it (P.P.); 2 Experimental Zooprophylactic Institute of Lombardy and Emilia Romagna, Via Modena 483, 44124 Ferrara, Italy; federica.lochiano@icloud.com (F.L.C.); silva.rubini@izsler.it (S.R.); 3 Department of Life Sciences and Biotechnology, Faculty of Medicine, Pharmacy and Prevention, Master Course in Cosmetic Science, University of Ferrara, Via L. Borsari 46, 44121 Ferrara, Italy; erika.baldini@unife.it

**Keywords:** carbamates-based drugs, oxamyl, GC/MS chromatography, poisoning, north Italy, companion animal, wildlife

## Abstract

Oxamyl is a highly toxic carbamate molecule with toxicological risk from contamination, used as an insecticide, nematicide, and acaricide on many field crops, vegetables, fruits, and ornamentals. Suspected poisoned animals and baits were collected between January 2018 and August 2021 from Lombardy and Emilia-Romagna regions and analyzed at the chemical toxicology laboratory of the Experimental Zooprophylactic Institute of Lombardy and Emilia-Romagna, located in Brescia. The analyses were carried out by an ion trap GC-MS system in 2467 suspected samples and showed the presence of oxamyl in 67 of these. In this study, we analyzed 47 (out of 67) positive baits: the provinces in which more cases have been recorded are Mantua, Ferrara, and Cremona, which overall had 72% of positivity. The nature of the analyzed samples was mostly corn (55.3%), followed by bird carcasses (19.1%), apples (14.8%), meatballs (2.1%), bread (2.1%), and other (8.5%). The use of oxamyl to produce poisoned baits is constantly increasing, proving that it must be considered as a public health risk for the possible consequences on target and non-target organisms, including humans.

## 1. Introduction

The carbamate group is a key structural motif in many approved drugs and prodrugs. There is an increasing use of carbamates in medicinal chemistry and many derivatives are specifically designed to make drug-target interactions through their carbamate moiety [1]. *N*-monomethyl carbamates, such as carbaryl, oxamyl, fenobucarb, and ethienocarb, are used as insecticides. *N*-dimethyl or ethyl methyl carbamates such as the natural alkaloid physostigmine, neostigmine, or rivastigmine that derive from physostigmine can also be used in medicine [2].

These compounds are AChE reversible inhibitors [3] by carbamylating the enzyme reactive site, leading to the accumulation of ACh at synapses, and to the same characteristic toxic manifestations (cholinergic toxidrome) described for organophosphorus insecticides.

However, beside their peculiar design, an ever-increasing use as pesticide is observed with foods contaminations. The Community Rapid Alert System for Food and Feed (RASFF) sent a notification (2020.2038 of 14/05/2020) for the presence, in a high concentration, of residues of the insecticide oxamyl (0.18 mg/kg—ppm) in a consignment of carrots from Italy. RASFF’s sampling was carried out on 6 May 2020, following a check carried out by the Swedish authorities, who seized the goods. The consignment of Italian carrots was destined for Sweden. The insecticide was present in the carrots at a value of 0.18 milligrams per kilo, 14 times higher than the permitted Maximum Residue Limit (MRL), which is set at 0.01 milligrams per kg-ppm (parts per million), a minimum detectable amount, making it an unauthorized product in practice [4].

Oxamyl is a carbamate used as a pesticide that comes in two forms: granulated and liquid. The granulated form has been banned in the United States where it is considered extremely dangerous for humans and animals if ingested, inhaled, or in contact with skin.

The discovery of poisoned baits is a public health problem, as it is a potential risk for the health of domestic and wild animals, but also for that of humans and the environment [5], thus representing a danger for both target and non-target organisms [6].

In Italy, to fight this phenomenon throughout the national territory, the Ministerial Ordinance of 18 December 2008, as amended, was issued, containing the “Rules on the prohibition of the use and possession of baits or poisoned baits” [7].

This procedure establishes that ‘it is forbidden for anyone to improperly use, prepare, mix and abandon baits and poisoned baits and mouthfuls or those containing toxic or harmful substances […], the possession, use and abandonment of any food prepared in such a way as to cause intoxication or injury to the person who ingests it’ (Art. 1, paragraph 1). In addition, ‘rodent-control and pest-control operations, carried out by specialized companies, shall be carried out in such a way as not to harm in any way people and other non-target animal species and shall be publicized by the companies themselves by means of notices displayed in the areas concerned […]’ (Art. 1, paragraph 3) [3,7].

Previous studies [8,9] have shown that different classes of pesticides are regularly used to produce poisoned baits: the most commonly reported causes of acute poisoning in Europe are insecticides (organophosphates and carbamates), rodenticides (anticoagulant rodenticides, zinc phosphide, bromethalin, strychnine), avicides (alpha-chloralose), and molluscicides (metaldehyde, methiocarb).

The high toxicity of carbamates results from their mechanism of action, which is based on the inhibition of AChE, resulting in prolonged overstimulation of the nervous system and skeletal muscles [10]. Typical clinical symptoms of carbamate intoxication are, therefore, excessive salivation accompanied by frequent urination and defecation, muscle tremors, and ataxia, which can rapidly develop into convulsions. Early diagnosis does not guarantee survival and death may occur within minutes of ingestion, due to respiratory failure caused by dysfunction of the diaphragm and intercostal muscles [11,12].

**Figure 1:**
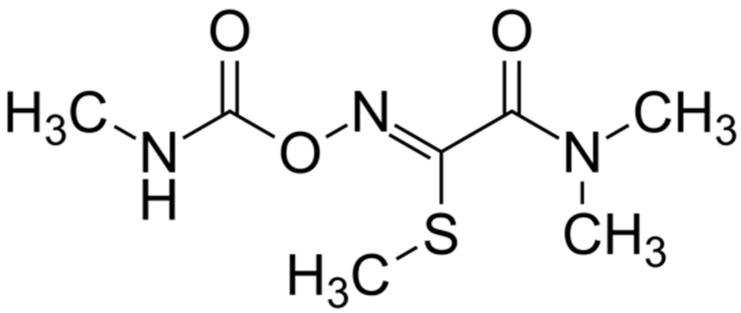
Oxamyl (methyl 2-(dimethylamino)-N-(methylcarbamoyloxy)-2-oxoethanimidothioate).

Within the carbamate family is oxamyl (methyl 2-(dimethylamino)-N-(methylcarbamoyloxy)-2-oxoethanimidothioate) (Figure 1), which occurs as a white, crystalline solid with a slight sulfurous odor [13].

It is used as an insecticide, nematicide, and acaricide on many crops, vegetables, fruits, and ornamentals such as apples, bananas, carrots, celery, citrus, cotton, cucumbers, eggplants, garlic, ginger, melon, onions, peanuts, pears, peppers, peppermint, pineapples, bananas, potatoes, pumpkins, soybeans, spearmint, squash, sweet potatoes, tobacco, tomatoes, and watermelons [13]. It can be applied directly to plants or to the soil surface [14].

In addition, when added with horse manure, sesame cake, or *Bacillus thuringesis*, it improves the growth response of eggplant and reduces the development of the nematode *Meloidogyne incognita* [15].

It has a high solubility in water (280 g/L at 25 °C), a very low soil absorption coefficient (Koc = 25), where it is degraded by hydrolysis into its non-toxic metabolite, methyl N-hydroxy-N′, N′-dimethyl-thioxamimidate [16]. The formulations are stable and, in fact, the aqueous solution decomposes slowly: it has a half-life of >31 days at pH 5.8 days at pH 7, and 3 h at pH 9 [17]. Aeration, sunlight, alkalinity, and higher temperatures increase the rate of decomposition [18].

Results of previous studies show that its presence in soils induces changes in the abundance of microorganisms that degrade it and in the diversity of the soil bacterial community [19]. Furthermore, Osman et al. [20] showed that the biodegradation rate of oxamyl in contact with animal manure is 5–8 times higher than without manure, which can be attributed to the presence of microorganisms in animal manure.

The formulations currently on the market in Italy can be consulted in the database of plant protection products, which the Ministry of Health makes public and keeps up to date: generally marketed under the name Vydate, oxamyl is available in granular form or as a soluble concentrate [21].

Carbamate pesticides are used intensively as pesticides in agriculture due to their broad spectrum of action [20] and are, therefore, reported to be one of the main causes of the intentional or accidental poisoning of animals [12].

The onset of symptoms following exposure to carbamates depends on the dose, route of exposure, and type of carbamate involved [22,23].

Acute oxamyl exposure leads to a cholinergic seizure, with more rapid effects following ingestion and inhalation than dermal exposure. Signs and symptoms may include increased salivation, lacrimation, bradycardia, sweating, spontaneous defecation and urination, miosis, blurred vision, tremors, muscle twitching, mental confusion, convulsions, and coma. Gastrointestinal symptoms include abdominal pain, diarrhea, nausea, and vomiting. Dyspnoea and pulmonary edema may also occur [24].

It is a highly toxic compound, so much so that the EFSA’s recommended risk classification places it within Class IB: highly hazardous [25]. It is, therefore, only permitted to be used by suitably trained personnel with a specific license [26].

The main reasons for deliberate poisoning are intolerance towards wild and domestic animals [27], the economic interests of hunters and truffle hunters, the application of urban hygiene measures, and the desire to protect human activities such as agriculture [6,28].

When a bait is found, the competent health authority draws up a report of suspected poisoning, giving all the anamnestic information needed to direct the search and identification of the toxic substance contained. Then, the bait and the relevant report form are sent to the Experimental Zooprophylactic Institutes competent for the territory, as reference laboratories for carrying out the inspection of the baits and the toxicological analyses necessary to confirm the diagnostic suspicions [29].

The aim of this work is to examine the presence of oxamyl in poisoned baits, organic liquids, and animal carcasses sent by the various territorial offices of the Experimental Zooprophylactic Institute of Lombardy and Emilia-Romagna to the toxicology chemistry department in Brescia, in the period between January 2018 and August 2021.

## 2. Materials and Methods

### 2.1. Study Area

The study area can be divided into:

- Lombardy (Lat 45.67° North; Long 9.5° East) and Emilia-Romagna (Lat 44.75° North; Long 11° East), which are two regions located in northern Italy, with a total area of 46,308 km^2^ and 14.4 million inhabitants [30];
- Lombardy borders Switzerland to the north, and Trentino-Alto Adige and Veneto to the east;
- Piedmont to the west and Emilia-Romagna to the south. Emilia-Romagna is bordered to the north by Lombardy and Veneto, to the west by Lombardy and Piedmont, to the south by Liguria, Tuscany, Marche, and the Republic of San Marino, and to the east by the Adriatic Sea.

Overall, three zones can be distinguished: mountains, hills, and plains, as well as large natural wetlands (Comacchio Valleys and Po Delta) located in the eastern part of Emilia-Romagna [31].

Almost half of the territory (48%) is characterized by the presence of a large plain, the Pianura Padana, a flat region of fluvial origin, with fertile conditions for cultivation and intensive livestock activities [32].

Lombardy, with more than 50,000 farms and 8000 food companies, is at the top of the Italian agricultural sector, and whose activities cover 69% of the national territory with traditional products and quality agri-food productions [33].

In addition, in Emilia-Romagna, agricultural land covers about 68% of the regional surface and includes areas with high agricultural yields in the lowland part, followed by forest and semi-natural areas (25%), artificial surfaces (5%), water bodies (1%), and wetlands (<1%) [34].

Wild animals are also relevant in the pre-Alpine area and in the wooded and shrubland area of the hills and high plains [31].

In this context, the presence of pesticides in the environment could be related to production activities.

### 2.2. Laboratory Analysis

The determination of carbamate oxamyl was performed through a semiquantitative GC-MS method, as described in the work of Chiari et al. [31] and Bertero et al. [35], using gas chromatography coupled with mass detector (GC/MS).

A typical 30 g of sample was weighed, added to 30 g of dry sodium sulfate, and extracted with acetone RPE for analysis (100 mL) by shaking for 1 h in a blender. After filtration on paper, 2 mL of filtrate was collected and the solvent was stripped out under a gentle nitrogen flow at 40 °C. The residue was reconstituted with 2 mL of acetone Chromasolv for pesticide’s residue analysis and injected (1 microliter) into an ion trap GC-MS system (Varian 240-MS ion-trap equipped with a Varian 450-GC, purchased from Agilent Technologies USA, Santa Clara, CA, USA). The chromatographic separation was carried out by a capillary column DB 5MS (30 m, 0.25 mm i.d., 0.25 mm film, Agilent Technologies USA) using helium as gas carrier. The detection was performed by the ion-trap mass analyzer: full mass spectrum of the target compound was acquired, providing the best identification.

The limit of instrumental detectability was 25 pg (S/N = 3). The limit of quantification of the method was 0.1 mg/kg (S/N = 5).

Acetone RPE (purity ≥ 99.8%) was purchased from Carlo Erba Reagents (Milan, Italy). Acetone Chromasolv for Pesticides Residue Analysis was purchased from Sigma-Aldrich (Milan, Italy). Oxamyl standard (purity 99.5%) was purchased from LGC Dr. Ehrenstorfer (Augsburg, Germany).

## 3. Results

During the period under review, the toxicology laboratory received 2488 samples for oxamyl. Of these, 2467 samples were analyzed and oxamyl was detected over the LOQ in 67 (2.7%). The semiquantitative results range from 0.3 mg/kg to 70,888 mg/kg, with an average value of 2531 mg/kg.

Two-thirds of the positive samples (47) were baits, from seven of the twenty provinces in the whole study area. Twenty-one samples (0.8%) could not be tested because they were unsuitable or insufficient.

**Figure 2:**
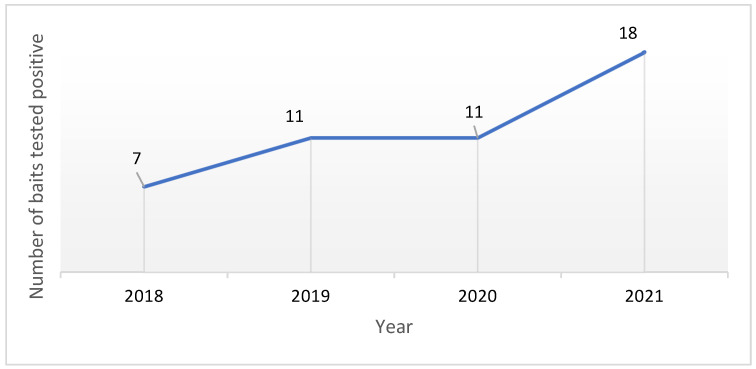
Number of baits tested positive, broken down by year.

The 47 positives found in the baits are distributed over time as follows: seven in 2018, eleven in 2019 and 2020, and eighteen in January–August 2021 (Figure 2).

**Figure 3:**
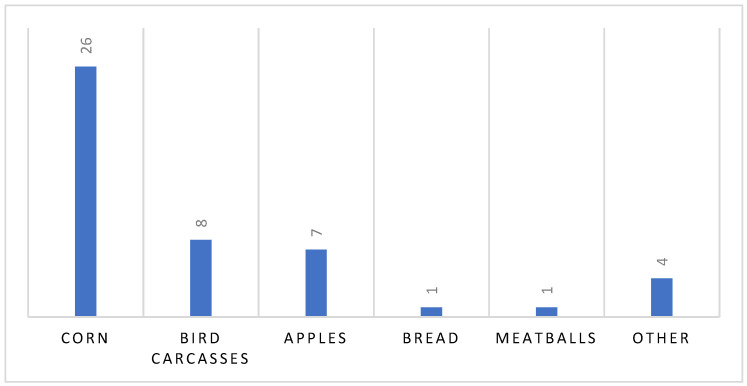
Type and number of positive matrices.

In terms of the nature of the samples analyzed, the absolute number and percentage of each is given below: maize (n = 26, 55.3%), poultry carcasses (n = 8, 17%), apples (n = 7, 14.8%), bread (n = 1, 2.1%), meatballs (n = 1, 2.1%), other (n = 4, 8.5%) (Figure 3).

**Figure 4:**
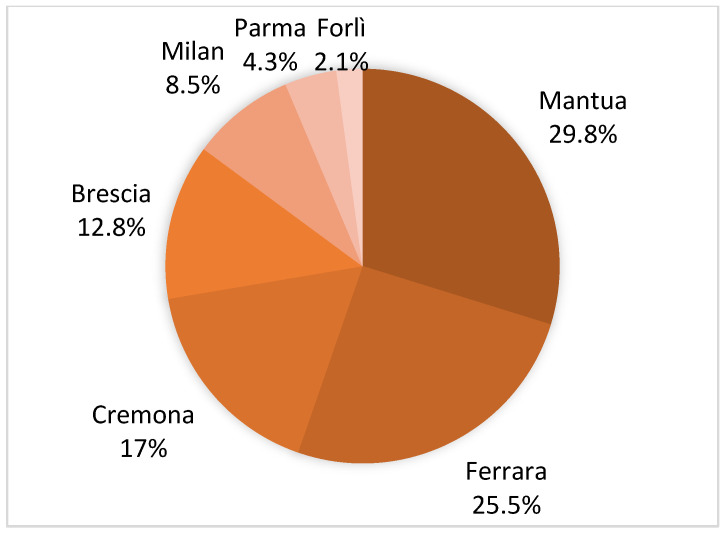
Number of positives, in percentage values, for each province.

The study area covered the whole of Lombardy and Emilia-Romagna, but only in some of these provinces were positives reported in the baits tested (Figure 4).

**Figure 5:**
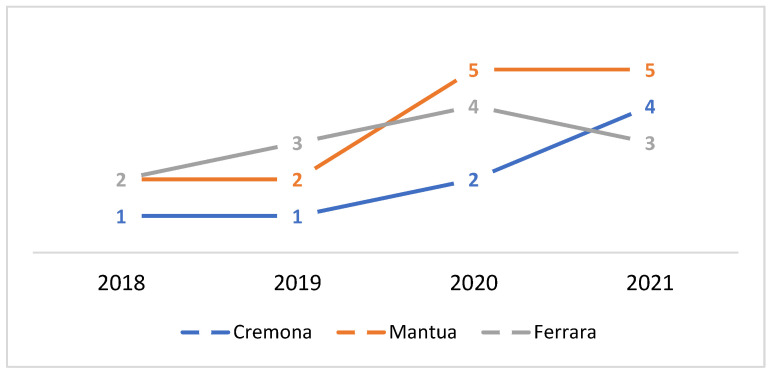
Number of cases per year in the first 3 provinces.

In particular, the provinces of Mantua, Ferrara, and Cremona together recorded 72% of positivities, with the number of annual reports increasing steadily since 2018 (Figure 5).

Finally, examining the nature of the matrices found to be positive in each of the provinces, the following results were obtained:

- Mantua: thirteen samples of maize and one of gastrointestinal content;
- Ferrara: six samples of apples, two samples of maize, three of gastrointestinal contents, and one chicken carcass;
- Cremona: six samples of maize, one sample of bread, and one meatball;
- Brescia: six chicken carcasses and one maize sample;
- Milan: four samples of maize;
- Parma: two poultry carcasses;
- Forlì: one apple sample.

**Figure 6:**
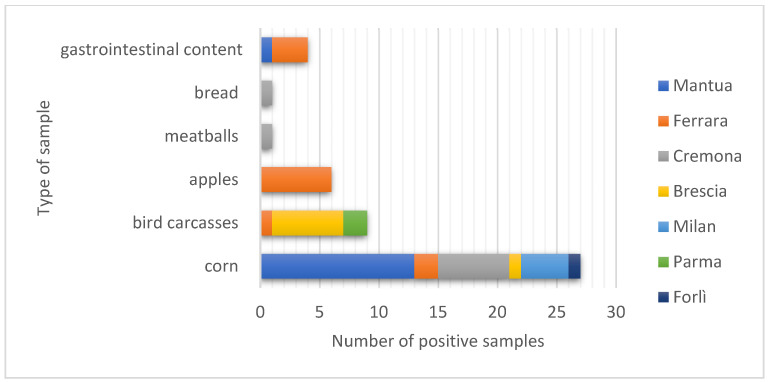
Number of positive samples.

Baits containing maize were the most frequently reported in Lombardy, especially in the provinces of Mantua, Cremona, and Milan (Figure 6).

## 4. Discussion

The effects of oxamyl exposure have been studied in terrestrial and aquatic organisms, following both acute and chronic exposures.

The molecule is highly toxic when administered as a single oral dose: Kennedy [36] calculated its LD50 to be 2.5 to 3.1 mg/kg in fasted rats, 2.3 to 3.3 mg/kg in fasted mice, and 7 mg/kg in guinea pigs. One beagle dog given 30 mg/kg died, while 15 mg/kg was non-lethal. Clinical signs of cholinesterase inhibition were observed in all species, but cholinesterase activity was reduced in rats treated with a single oral dose.

When administered by intraperitoneal injection, it was found to be highly toxic to rats, mice, and guinea pigs. It is also mildly irritating to the eyes. With regard to contact reactions, oxamyl produced mild skin irritation and the toxicity of skin absorption in rats (LD50 is >1200 mg/kg) and rabbits (740 mg/kg) was relatively high, suggesting limited absorption. Oxamyl is highly toxic by inhalation with the 1-h LC50 value in rats being 0.17 mg/L (male) and 0.12 mg/L (female).

Again, Anguiano et al. [37] demonstrated the effects of exposure to orphanophosphate and carbamate on the inhibition of AChE activity in the gill tissue of Crassostrea gigas, using dichlorvos, carbofuran, and oxamyl.

Similar results were obtained in starlings (*Sturnus vulgaris*) [38] and honeybees [39].

With regard to chronic toxicity, studies in rats, mice, and rabbits suggest that oxamyl has no carcinogenic, teratogenic, or embryotoxic effects. However, in one- and three-generation breeding studies, litter size was seen to be slightly lower in rats fed oxamyl at 100 or 150 ppm than in those fed 50 ppm. No evidence of a teratogenic response was observed in the rat, although a reduction in the rate of weight gain was observed in pregnant rats fed oxamyl at 100 ppm or more [40].

In the case of suspected poisoning, when the local IZS receives a sample, it carries out an inspection of the material received in order to target the search for certain categories of molecules. The suspicion of the presence of oxamyl arises from the fact that, although it is a colorless substance, in commercial products, dyes are added to give the bait a characteristic color; for example, the commercial product Vydate has tartrazine added, which gives it a green color, the same color as the artificially contaminated bait. Therefore, the color becomes a danger indicator.

The decision to use apples as bait is probably due to one of the animal species for which the bait is intended, namely the nutria (*Myocastor coypus*), a small, alien mammal that causes a great deal of damage to both crops and the ecosystem [41]. The use of oxamyl is in fact more frequent in rural areas where nutrias are very numerous.

This type of bait is used by farmers to combat wildlife. The baits or bites do not recognize specific targets, but can be ingested by various non-target species, can contaminate crops, and can contaminate water through runoff. In practice they constitute real ecological bombs that will create harm to human health in the long run.

**Figure 7:**
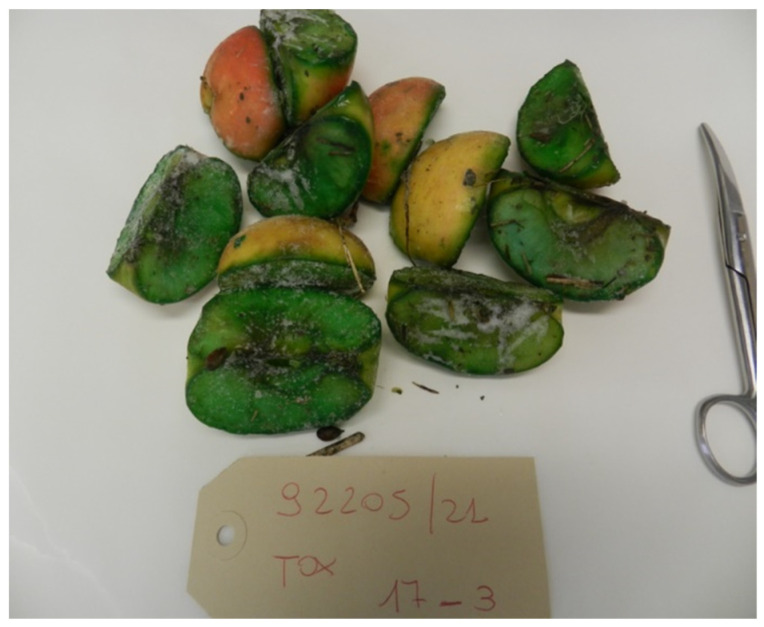
Typically deep green apples.

**Figure 8:**
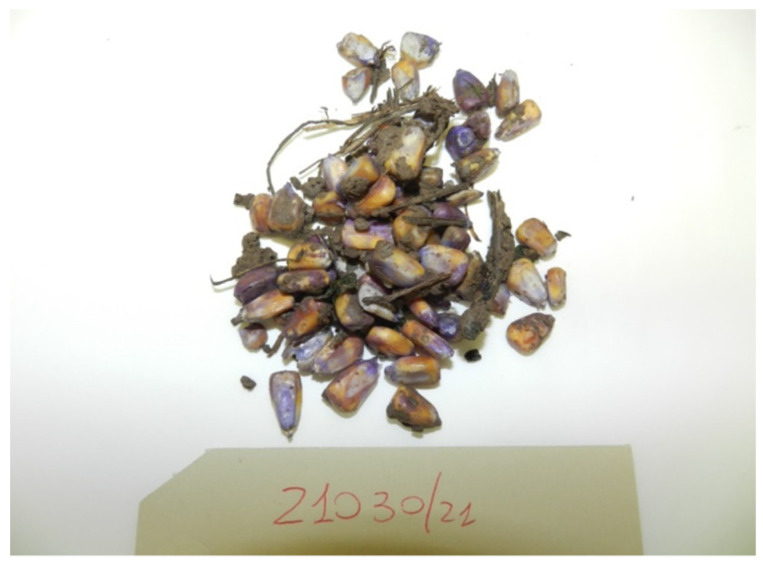
Violet corn.

**Figure 9:**
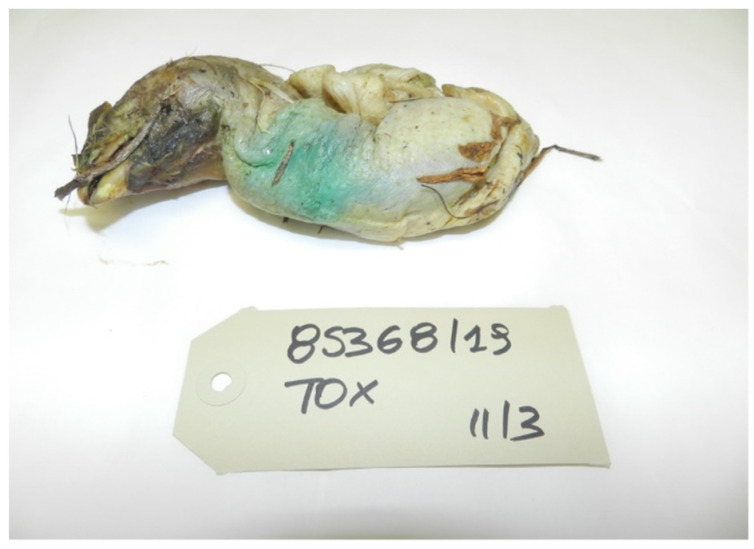
Chicken carcass with blue–green coloring.

Below are the most frequently received bait categories (Figure 7, Figure 8 and Figure 9).

Analyzing the results shown in Figure 2, it can be seen that the trend in the number of reports over the time period under study is steadily increasing. There has also been an increase in the number of provinces from which reports are received: from four provinces in 2018 to six provinces in the first months of 2021. It should also be borne in mind that it cannot, therefore, be ruled out that further positives will occur in the remaining four months of 2021.

As mentioned above, most of the reports came from the provinces of Mantua, Ferrara, and Cremona, which together accounted for 72.3% of the positive findings. In these provinces, with regard to the matrices tested, the majority are apples and maize, the latter being almost exclusive to the province of Mantua. This could be related to the fact that their production is more consistent in that area.

In the category ‘other’, all baits other than those described were included: these are gastrointestinal contents. These were either delivered directly to the laboratory or taken during necropsy.

The distribution of suspected and confirmed cases of poisoning does not show a typical seasonal pattern, which would justify deliberate poisoning by the buyer and holder of the product rather than accidental poisoning related to the use of oxamyl in different crops, depending on the different agricultural activity during the year.

Our data show that it is desirable for the problem that the competent authorities elaborate specific prevention plans for the containment of agricultural pests so as not to let the individual provide for themselves. The possibility that, through runoff, high concentrations of harmful substances reach the deepest strata of the soil and groundwater means that these can become real ecological bombs that, in the long term, will create damage to human health.

Although the term “One Health” (OH) was coined recently, the concept related to it has long been recognized both nationally and globally. The only way forward is the realization that we live within a system of which people, animals, plants, and generally, the environment in which we are all immersed are a part. So, there are not only individuals and communities, but there is also not only the human species to be preserved: the health of the planet and all its inhabitants must be given equal dignity if we are to create a sustainable, resilient, and enduring ecosystem. We are all elements of one system, in which the health of each human, animal, or environmental element is closely interdependent on that of the others.

## 5. Conclusions

The data compiled in this study provide a picture of oxamyl poisoning in Lombardy and Emilia-Romagna. To date, numerous toxicity studies on various animal species are available in the literature, but data on the presence of oxamyl throughout the country are scarce. Our analysis confirms that, in spite of numerous restrictions and bans on the use of toxic pesticides, they are still widely used in the preparation of poisoned baits and that insecticides are one of the most commonly used categories in this respect.

In addition, there has been a steady increase in the number of cases reported during the time period covered by this work. This is all the more worrying given that only the first eight months of 2021 were taken into account.

This demonstrates the need to strengthen monitoring and control activities by health authorities, which would otherwise lead to an underestimation of the actual cases, and to inform about the risks to human and animal health.

In this regard, and as our study shows, the type of bait, together with the particular coloring, must give priority to the search for oxamyl.

## Data Availability

Not applicable.
